# Constructing intervertebral disc degeneration animal model: A review of current models

**DOI:** 10.3389/fsurg.2022.1089244

**Published:** 2023-03-10

**Authors:** Tongzhou Liang, Bo Gao, Jinlang Zhou, Xianjian Qiu, Jincheng Qiu, Taiqiu Chen, Yanfang Liang, Wenjie Gao, Xuemei Qiu, Youxi Lin

**Affiliations:** ^1^ Department of Orthopedic Surgery, Sun Yat-Sen Memorial Hospital of Sun Yat-Sen University, Guangzhou, China; ^2^ Department of Operating Theater, Sun Yat-Sen Memorial Hospital of Sun Yat-Sen University, Guangzhou, China

**Keywords:** intervertebral disc degeneration, nucleus pulposus, intervertebral disc, animal model, surgery technique, orthopedics surgery

## Abstract

Low back pain is one of the top disorders that leads to disability and affects disability-adjusted life years (DALY) globally. Intervertebral disc degeneration (IDD) and subsequent discogenic pain composed major causes of low back pain. Recent studies have identified several important risk factors contributing to IDD's development, such as inflammation, mechanical imbalance, and aging. Based on these etiology findings, three categories of animal models for inducing IDD are developed: the damage-induced model, the mechanical model, and the spontaneous model. These models are essential measures in studying the natural history of IDD and finding the possible therapeutic target against IDD. In this review, we will discuss the technical details of these models, the duration between model establishment, the occurrence of observable degeneration, and the potential in different study ranges. In promoting future research for IDD, each animal model should examine its concordance with natural IDD pathogenesis in humans. We hope this review can enhance the understanding and proper use of multiple animal models, which may attract more attention to this disease and contribute to translation research.

## 1. Introduction

Low back pain (LBP) is one of the most common disorders affecting elder and middle-aged persons. It has been estimated that low back pain is the fourth most prevalent disease that causes disability worldwide (1). In the last 30 years, DALYs of low back pain has increased by approximately 33% (2). In the US, the total cost of LBP is 7.4 billion US dollars in 2008 (3). Given the high prevalence and high cost of LBP, it is urgent to search for the pathogenesis of LBP and develop treatments for alleviating LBP (4).

Intervertebral disc degeneration (IDD) is one of the major causes of LBP (5–7). Approximately 40% of LBP presented with the feature of IDD (8). The Intervertebral disc consists of three major histological distinct components: annulus ﬁbrosus (AF), nucleus pulposus (NP), and cartilage endplate (EP). In undegenerated intervertebral disc, NP was surrounded by AF with CEP covering the interface between AF and bony vertebrae. Known risk factors for IDD include excessive mechanical loading, obesity, spine imbalance, diabetes mellitus, and genetic predisposition (9–13). When the process of IDD commences, internal and external stimuli triggers inflammation and oxidative stress. The overproduction of inflammatory mediators, such as tumor tumor necrosis factor-alpha (TNF-α), interleukin 1-beta (IL-1β), and interleukin 6 (IL-6), led to increased expression of extracellular matrix (ECM) degradation enzymes (14, 15). The overproduction of ECM degradation enzymes leads to loss of collagen type II and aggrecan in NP and subsequently compromises the water-retaining ability (16). Loss of water and ECM components leads to biomechanical changes in the intervertebral disc and exacerbates IDD (17, 18).

**Figure 1:**
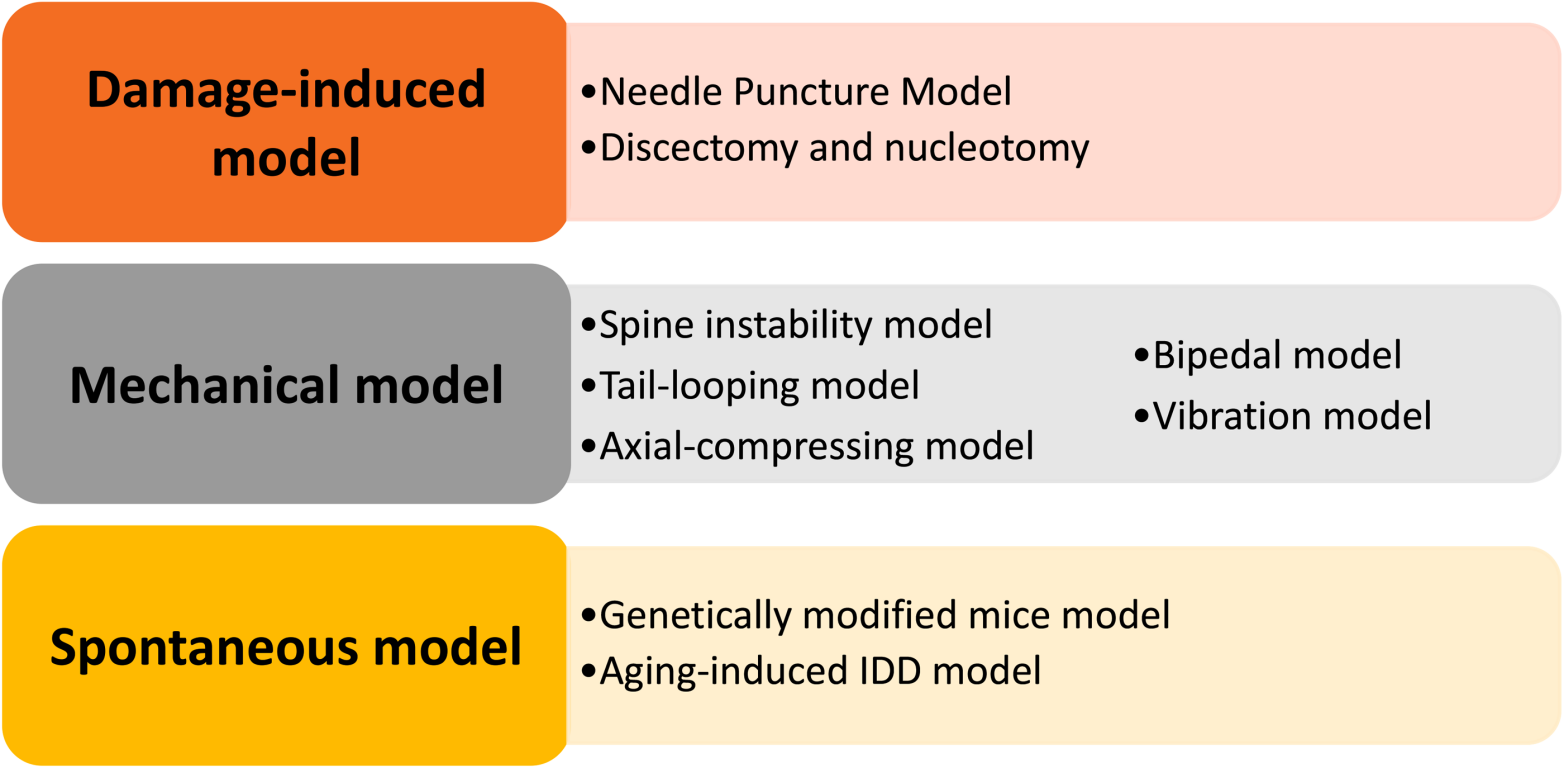
Summary of the animal models demonstrated in this review, including the damage-induced, mechanical, and spontaneous models.

It is of great significance to develop and utilize IDD animal models to understand the pathogenesis mechanism and test novel treatments. We elaborate on currently available animal models and provide an overview of the utility of these models. In this review, we tried to present the advantages and disadvantages of these models, discuss the duration of constructing these models, and include some necessary technical details of model construction (Figure 1). Finally, we hope this review will contribute to the appropriate selection of IDD models and promote the development of new therapeutic strategies.

## 2. Methods for constructing IDD animal model

### 2.1. Damage-induced model

#### 2.1.1. Needle puncture model

The needle puncture model was established through puncture of the intervertebral disc from either the posterior or anterior direction. This model is most commonly used in small animals, including rats, mice, and rabbits. However, needle puncture is also applicable in establishing the IDD model on larger animals like dog (19), sheep (20), bovine (21), and rhesus monkeys (22). The needle puncture model is easy to install by inserting the needle into AF without disrupting NP. The insertion depth can be determined by radiography monitoring or the length of the needle emerged. After insertion, the needles are usually placed in the disc for a period of 30 s to 1 min (23, 24). A proportion of studies rotated the needle for 180°–360° before being placed in the disc (25, 26).

Different diameters of needles are used to induce the IDD animal model. Chen et al. inserted a 21G needle into the AF of rats and IDD was observed in the corresponding level 4 weeks post-operation (27). Our experiments also confirmed the successful induction of IDD histologically and radiographically four weeks after 21G needle insertion (28). Smaller and larger needles are also demonstrated to induce IDD in rats post-surgery. Issy et al. showed that 30G needles insertion can cause IDD 6 weeks post-operative (29). Matta et al. performed the puncture model with 32G needles, and the animals were euthanized ten weeks after modeling (30). In rats inserted with larger needles (20G), the occurrence of IDD is more rapid than minor needle insertion as the histological IDD was observed one week after injury (31, 32). Masuda et al. compared the histological damage of needle puncture using 16G, 18G, and 21G needles in the rabbit model of IDD. Generally, these studies suggested that the radiographic and histologic damage is more severe in mice punctured with a larger needle (33). In large animals, a larger size of needles is needed to induce the IDD model. Tellegen et al. inserted 18G needles on the AF of dogs under the monitoring of intra-operative fluoroscopy. These studies suggested that the development of IDD is closed related to the diameter of needles and choosing the appropriate needle and sampling time are crucial to the conclusion.

Punctures in both lumbar (L) discs and coccygeal (Co) discs can induce IDD. In the lumbar disc puncture model, skin incisions are needed to expose the lumbar disc. Kim et al. performed a right hemilaminectomy to expose the L5/6 disc and inserted a needle. Von Frey test, Basso-Beattie-Bresnahan scale, and the horizontal ladder test found that the rats emerged behavior in response to pain as early as 1-week post-surgery (34). Coccygeal disc puncture can be performed with or without fluoroscopy (35, 36). Co5/6, Co6/7, Co7/8, Co8/9, and Co9/10 levels are usually selected for the IDD modeling (37, 38). Isa et al. established the IDD model by puncturing Co4/5 and Co5/6 discs and then investigated the pain response in the ventral base of the tail by Hargreaves test, von Frey test, and tail-flick test (39). Interestingly, lumbar discs and coccygeal discs puncture may represent the different modeling scenarios. In evaluating the behavioral parameters of IDD, lumbar disc puncture seems more resemble with IDD in patients since it may induce both leg and back pain. The coccygeal discs puncture models are easier to perform and may benefit the research for alleviating the disc degeneration process.

Needle puncture combined with intradiscal injection of reagents also represents a method for constructing the IDD model. Since pro-inflammatory factors contribute to the onset of IDD (5), injection of pro-inflammatory factors such as TNF-α and IL-1β into the intervertebral disc can induce IDD in different animal models (34, 40). Notably, the pain response to needle puncture is associated with the expression level of pro-inflammatory cytokines in the dorsal root ganglion (41). Norcross et al. compared injection of chondroitinase ABS or phosphate-buffered saline into the intervertebral disc of rats. Disc height and histological examination showed that chondroitinase injection leads to the observable degeneration on day 14 of experiment (42). Complete Freund's adjuvant (CFA), a tissue destruction reagent, was injected into to intervertebral disc to induce IDD (43). The rats were subjected to the behavioral test and found CFA injection successfully induced back pain and inflammatory factors accumulation (44). Wei et al. injected pingyangmycin or bleomycin into the subchondral bone adjacent to the lumbar intervertebral disc of rhesus monkeys, and degeneration was observed by MRI 3 months after injection (45, 46).

#### 2.1.2. Discectomy and nucleotomy model

Discectomy is the standard surgical procedure for treating intervertebral disc herniation caused by IDD (47). Discectomy can relieve the nerve root compression by disc herniation, but the loss of NP tissue may cause the subsequent collapse of the intervertebral space (48). Therefore, discectomy model is suitable for studying disc-healing therapy, especially implants or biomaterials. The discectomy was performed on multiple animals, including rats (49), rabbits (50), sheep (51), pigs (52), and bovine (53). Since many studies suggested that goats and sheep possess similar biomechanical properties that are similar and comparable to the human spine, both animals are considered to be suitable for investigating spine mechanical properties (54, 55). Sloan et al. performed the discectomy in 3-4-year-old Finn sheep by performing a 3 × 10 mm annulotomy and then removing 200 mg of NP tissue (56). The intervertebral discs were subjected to histological examination six weeks after surgery. NP heterogeneity, AF lesions, and increased proteoglycan staining were observed in AF (56). Oehme et al. investigated a mini-invasive approach in sheep by making a 3 × 5 mm rectangular annular incision on AF using an 11-blade scalpel (57). A mixture of NF and AF tissue weighing 200 mg was removed.

Nucleuotomy refers to the partial excision of NP tissue with little disturbance of AF structure (58). Schwan et al. introduced a novel surgical approach to performing nucleotomy (59). A skin incision was made, and the corresponding disc level was determined with x-ray fluoroscopy. The discs were punctured with a straight awl through the whole layer of AF, and surgical channels were created. A 12 cm long rongeur was inserted through the tunnel, and approximately 0.15 cm^3^ of NP tissue was removed (59, 60). Partial nucleotomy resulted in the loss of disc height six weeks after surgery. Takeoka et al. performed percutaneous nucleotomy in rats according to the method by Nishimura et al., and loss of disc height was observed as early as seven days post-surgery (61).

### 2.2. Mechanical model

#### 2.2.1. Spine instability model

In 1991, Miyamoto et al. proposed a model of constructing cervical spondylosis by surgically induced spine instability (62). The posterior paravertebral muscles were separated, and the cervical, thoracic and lumbar spine was exposed. Then the spinous processes and attached supraspinous and interspinous ligaments were resected. The model was commonly used in constructing the IDD model in the lumbar spine and is therefore referred to lumbar spine instability (LSI) model. Zheng et al. applied the L3–L4 LSI to investigate the contributory role of parathyroid hormone in maintaining intervertebral disc homeostasis (63). Xue et al. further exploited the model to examine the role of skeletal interoception in causing EP degeneration and spinal-associated pain (64). Recently, Liu et al. demonstrated that the LSI model leads to spinal hypersensitivity in DRG, which explains the pain caused by IDD (65).

As for constructing the caudal spine instability model, Bian et al. stapped through the full depth of the Co7/8 AF, and then removed the NP (66). The adjacent Co8/9 intervertebral disc was subjected to histological analysis four weeks post-surgery and confirmed the successful establishment of the IDD model. Another study by the same group also made an incision into the whole layer of AF and performed NP removal to induce IDD. The bony EP was then analyzed and found that CSI leads to bony EP porosity of the same level (67).

#### 2.2.2. Tail-looping model

Clinical observations suggested spinal deformities such as adolescent idiopathic scoliosis (AIS) and Scheuermann's disease are associated with IDD (68, 69). The spine deformity alters the normal pattern of force distribution and undermines the mechanical property. Tail-looping model is a novel method to construct the IDD model by creating force imbalance within the intervertebral disc. Saikai et al. looped the tail of mice and made fixation between Co5 and Co13 vertebrae with 0.8-mm stainless steel wire (70). The extra vertebrae were excised. The NP of Co7/8, and Co8/9 discs were aspirated to generate more severe degeneration. In this model, the Co2/3 and Co3/4 discs were selected as control discs, while Co10/11 and Co11/12 discs were chosen as mildly degeneration discs. The researchers demonstrated that histological severity correlates with previous treatment, and degeneration occurred as early as eight weeks after looping (70). Nakamichi et al. established the tail-looping induced IDD model by joining Co2 and Co9 vertebrae together (71). In this study, outer AF was removed, and the role of Mohawk-induced AF regeneration was explored. Further, Huang et al. modified the looping method by tying the tail with thin wire instead of stitching the vertebrae. The model was successfully constructed after two months of fixation and continued with the adenovirus treatment for one month before sample collection (72).

#### 2.2.3. Axial-compressing external fixation devices

Under the condition of compression and angulation, the intervertebral discs may become narrowed and stiffer (73). MacLean et al. annexed external rings to adjacent levels of intervertebral discs by inserting 0.5 mm pins transfixing the vertebral bodies percutaneously (74). Stokes et al. modified the method by installing rings either parallel to each other or with an angle of 15-degree (75). Their results showed that 15-degree angulation plus compression yielded greater disc space loss. Hirata et al. exerted temporary static compression using an Ilizarov-type apparatus with springs between Co8 and Co10 (76). An axial force of 1.3 MPa was exerted and subsequent analysis found that the compression reproduced different stages of degeneration. The same compression pressure was adopted by other studies (77, 78), suggesting this pressure may be the appropriate pressure for constructing model. In a recent study, Ji et al. developed a novel device by inserting Kirschner wire into Co8 and Co10 vertebral bodies (79). Then the tail was bent for 40° and springs are used to exert 1.8N and 4.5N of force on Co8/9 and Co9/10 intervertebral disc. Pfirrmann grades and histological examinations revealed the occurrence of IDD two weeks after surgery. The severity of degeneration correlates positively with the force exerted (79).

In addition to constructing the IDD model on a histological and radiographic level, the axial-compressing external fixation devices can simulate pain caused by IDD. Miyagi et al. used both the compression model and needle puncture model to establish IDD in rats and found that the pro-inflammatory factors were elevated (80). Moreover, the positive labeling of calcitonin gene-related peptide (CGRP) neurons increased, suggesting a potential mechanism IDD leads to low back pain (80). Since many studies have shown neurogenic factors like brain-derived neurotrophic factor (BDNF), nerve growth factor (NGF), and CGRP and closely related to discogenic pain of IDD (81, 82), the compression model is applicable in pain-related phenomenon.

#### 2.2.4. Vibration model

High-frequency, low-amplitude whole-body vibration (WBV) is a common physical therapy in some disorders. Notably, WBV has been used as an adjuvant treatment for osteoporosis, muscle weakness, and low back pain (83, 84). However, it remains controversial whether WBV retarded the progression of IDD. Clinical observations found that workers exposed to occupational vibration are susceptible to IDD (85). Studies have linked vibration exposure with increased matrix metalloproteinase and decreased ECM (86), suggesting vibration is a potential risk for IDD progression. In a study by McCann et al., they applied clinically used vibration frequency (45 Hz with peak acceleration at 0.3 g for 30 min per day and 5 days per week) on mice for four weeks (87). The morphologic grade was analyzed and confirmed the IDD occurrence, especially characterized by AF degeneration. Furthermore, they found that 4-week WBV followed by 4-week cessation did not reverse the IDD in mice, suggesting the damage is permanent (88). Zeeman et al. demonstrated that 8 Hz and 15 Hz WBV is associated with long-lasting cervical and lumbar pain in rats (89), indicating the WBV model may also be useful in pain-related research. Although it is now known that WBV contributes to the development of IDD and IDD-related pain, the ideal vibration mode (time, frequency, orientation) to induce IDD still needs more investigation (90).

#### 2.2.5. Bipedal animal model

The bipedal animal model was established by forelimb amputation in rodents. After forelimb amputation, a forced bipedal stance mimics the bipedal gait of human(91). Liang et al. performed amputation surgery on 1-month-old male rats and the rats were kept in custom-made cages to force them to stand in an upright position (92). The rat was kept for 5 months and 7 months before histological analysis. Loss of cervical disc height was observed in the amputation surgery group five months after surgery, and the height loss was more severe seven months after surgery. The down-regulation of Col2a1 and aggrecan was also observed and may decrease anti-compression capacity (92). Using the bipedal rat model, Liu et al. discovered ligustrazine attenuate cartilage EP hypertrophy, a characteristic of IDD, within an observation period of 9 months (93). Kong et al. Found the myocardin-related transcription factor A (MRTF-A) inhibitor CCG-1423 attenuated IDD progression over six months (94). Although the bipedal animal model mimics the upright posture similar to human beings, this model may take as long as six months to gain a histologically observable degeneration. Another concern that hampers the application of this model is animal welfare since amputation surgery causes trauma and alters the feeding habits of animals (91).

In addition to forelimb amputation, bipedal models are modified to yield better potency. Liang et al. performed both brachial plexus rhizotomy and tail amputation on 4-weeks-old female rats (95). The rats were euthanized six weeks post-surgery and lumbar discs were dissected (L1–S1) and subjected to qPCR analysis. Data showed the loss of ECM matrix in six weeks post-surgery, suggesting the efficacy of this model (95). Recently, Ao et al. developed a novel bipedal model utilizing the water-escape nature of rodents without amputation surgery (96). The mice were kept in a chamber with a 5 mm depth of water on the bottom of the chamber. The mice were kept in the chamber for 6 h each day and were allowed to access water and food freely for 2 h. Because of the water-escape nature, the mice are more likely to keep an upright position in the chamber. Degeneration of the facet joint and the intervertebral disc was observed 6-week after treatment (96). Lao et al. developed a hot plate cage to exert accumulated spinal axial force on mice's spine (97). The mice were placed on the 50 °C hot plate for 15 min per day and were forced to jump before returning to the regular cage. IDD was observed one-month post-modeling and progressed more severely in 3-months of observation (97).

### 2.3. Spontaneous model

#### 2.3.1. Genetically modified mice model

Certain gene deficiency impairs intervertebral disc metabolic homeostasis and structural integrity. Secreted protein acidic and rich in cysteine (SPARC) is a matricellular protein involved in the pathogenesis of IDD (98). The expression level of SPARC decreased with aging and intervertebral disc degeneration. Moreover, it has been demonstrated that SPARC deletion accelerates IDD in mice (98, 99). Histological analysis revealed that herniations of lower lumbar discs in SPARC-null mice occur as early as 14-month-old. Millecamps et al. discovered that in addition to typical IDD pathologic features, the SPARC-null mice also developed the feature of chronic back pain (100). The chronic back pain was characterized by hind paw sensitivity to mechanical and cold stimuli, intolerance to axial stretching, and motor impairment, which all implied nerve root impairment. More studies confirmed the association between low back pain and IDD in the SPARC-null mice (101). In a study by Lee et al., the SPARC-null mice developed IDD and low back pain at 14 months (98). Krock et al. found that the SPARC-null mice presented with low back pain at a relatively young age of 7–9 months (102).

SM/J mice is a strain characterized by lacking cartilage regeneration ability (103). It is reasonable to propose the hypothesis that intervertebral disc homeostasis in SM/J mice may be disrupted. Choi et al. found that the cellularity and matrix components of SM/J mice are altered at a young age (104). Severe IDD was observed in 17-week-old SM/J mice, marked by increased apoptosis and collagen degradation. Moreover, the intervertebral disc of SM/J mice showed increased stiffness and the vertebral bone showed decreased bone quality (104). Zhang et al. compared LG/J mice, a mice strain characterized by a remarkable ability to heal after cartilage injury, with SM/J mice in spontaneous IDD (105). Their result suggested the potential use of combining LG/J mice and SM/J mice in the genetic and biological study of IDD. Study by Novais et al., demonstrated that SM/J mice have increased susceptibility to IDD. However, the same study found that the LG/J mice showed increased disc calcification and degeneration compared with the BL6 strain, which is inconsistent with research mentioned above (106).

Studies have identified many genes associated with IDD, and knockout of these genes may also replicate the phenotype of spontaneous IDD. For example, collagen type II, encoded by the Col2a1 gene, has been identified as the critical regulator in intervertebral discs embryonic development (107). Col2a1 knockout showed the feature of AF glycosaminoglycans loss and EP degeneration in 9-month-old mice (108). Deletion in other collagen encoding genes, including Col9a2 (109), and Col9a1 (110), also exhibited the feature of spontaneous IDD. Besides the extracellular matrix components, loss in other genes (e.g,. Smad3 (111), IL-1rn (112), Hif-1a (113), Apoe 114) may also contribute to IDD's pathogenesis (Table 1). However, because IDD has long been considered a heterogeneous disease with different etiology, the use of the gene-specific knockout mice model may be limited.

**Table 1:** Genetically modified or certain strain mice that exhibits features of early IDD.

Strain	Method of analysis	Observed onset of degeneration	Year
SPARC-null mice (99)	Histological analysis Radiographic analysis	14-month-old	2005
SPARC-null (100)	Behavioral assays Histological analysis	78-week-old	2015
SPARC-null mice (102)	Behavioral assays Radiographic analysis Biochemical tests: ELISA	7-month-old	2019
SPARC-null mice (115)	Biomechanical test	18-month-old	2020
SPARC-null mice (98)	Behavioral assays Biochemical tests: qPCR	14-month-old	2022
SM/J mice (104)	Histological analysis Biomechanical test	17-week-old	2018
SM/J mice (105)	Histological analysis Biochemical tests: proteomes	8-week-old	2018
SM/J mice (106)	Histological analysis Radiographic analysis: *μ*CT	6-month-old and 23-month-old	2020
LG/J mice (106)	Histological analysis Radiographic analysis: μCT	6-month-old and 23-month-old	2020
Bmal1 CKO (Col2a1^Cre^Bmal^fl/fl^) (116)	Histological analysis Radiographic analysis: x-ray	6-month-old and 12-month-old	2017
Skt^Gt/Gt^ (117)	Histological analysis	8-week-old	2006
Col IX KO (118)	Histological analysis Radiographic analysis: μCT	6-month-old and 10-month-old	2016
TonEBP-deficient (119)	Histological analysis Radiographic analysis: μCT	22-month-old	2020
ERCC1-deficient (120)	Histological analysis	20-week-old	2010
Il1rn KO (125)	Histological analysis Biochemical tests: qPCR	55-day-old and 155-day-old	2013
IL-1 KO (112)	Histological analysis Radiographic analysis: μCT	12-month-old and 20month-old	2019
MCT4 KO (122)	Histological analysis Radiographic analysis: μCT	8-month-old	2020
Sox9 CKO (Acan^CreERT2^Sox9^fl/fl^) (123)	Histological analysis Radiographic analysis: μCT	12-month-old	2020
Mkx KO (71)	Histological analysis Biochemical tests: qPCR & Western Blotting	12-month-old	2016
Tgfbr2 CKO (Acan^CreERT2^;Tgfbr2^fl/fl^) (124)	Histological analysis Radiographic analysis: x-ray	6-month-old and 12-month-old	2018
CCN2 CKO (Noto^Cre^; CCN2^fl/fl^) (125)	Histological analysis	12-month-old and 17-month-old	2013
FOXO1/3/4 CKO (Col2a1^Cre^; Foxo1^fl/fl^; Foxo3^fl/fl^; Foxo4^fl/fl^) (126)	Histological analysis	4-month-old and 6-month-old	2018
FOXO1/3/4 CKO (Acan^Cre^; Foxo1^fl/fl^; Foxo3^fl/fl^; Foxo4^fl/fl^) (126)	Histological analysis	6-month-old and 12-month-old	2018
Smad3 KO (111)	Histological analysis	30-day-old and 60-day-old	2009
Hif1*α* KO (Shh^Cre^; HIF1α^fl/fl^) (113)	Histological analysis	6-week-old and 12-week-old	2013
Kindlin2 CKO(Acan^CreERT2^;Kindlin-2^fl/fl^) (78)	Histological analysis Radiographic analysis: μCT	18-week-old	2022

#### 2.3.2. Aging-induced IDD model

In 1988, Silberberg demonstrated that the sand rat (*Psammomys obesus*), a small desert rodent, is susceptible to age-related IDD (127). The severity of IDD was correlated with greater age. Helen et al. examined the age-related IDD of sand rats in a more detailed manner. The intervertebral discs of younger (2–11.9 months) and older (12–25 months) animals were collected and subjected to histological analysis. Their results suggested that the cervical spine of both younger and older sand rats is more likely to develop osteophytes than the lumbar spine. Moreover, the occurrence of osteophytes correlates with the extrusion of the intervertebral disc (128).

Some previous studies have demonstrated that mice are less susceptible to age-related IDD, which may limit the application of this model to some extent. For example, Marfia et al. showed that half of the mice did not exhibit IDD *via* MRI analysis in 19 months (129). Ohnishi et al.explored the availability of age-related IDD model in mice by MRI analysis followed by Pfirrmann classification and histological analysis followed by classification proposed in this research (130). They analyzed the mice aged 6 months, 14 months, and 22 months with both Pfirrmann classification and histological classification and found the feature of IDD progressed with increased age. The 14-months-old mice exhibited mild IDD while the 22-months-old mice developed moderate to severe IDD, which suggested that at least a 14-month follow-up is required for age-related IDD in C57BL/6 mice (130). Aging alters IDD's metabolism in many aspects, such as elevated chondrocyte hypertrophy and loss of notochordal markers (131).

The age-related IDD mice model is also widely used in investigating factors associated with senescence and longevity. Lin et al. found that tenomodulin (Tnmd), an anti-angiogenic transmembrane glycoprotein, maintained the structural integrity and matrix gene expression in outer AF and NP (132). Loss of *Tnmd* gene leads to early-onset IDD in 6-month-old mice and the IDD progressed more severely in 18-month-old mice compared with wild-type mice. Novais et al. investigated the role of senolytic drugs in ameliorating age-related IDD and defined different age groups, namely healthy adult (6-month-old), middle-aged (14-month-old), aged (18-month-old), and old-aged (23-month-old) (133). The mice started senolysis treatment at 6,14 and 18 months and IDD was harvested at 23 months.

## 3. Discussions

Intervertebral disc degeneration is a disease with complex etiology and clinical heterogeneity. Therefore, it is hard to find an ideal animal model that mimics all the inherent pathophysiology of IDD. Among these pathophysiology changes, some features are considered extra important, including loss of extracellular matrix and proteoglycans, biomechanical property alternations, and increased cell death. Discogenic pain is not necessarily associated with the severity of IDD (134), but the pain is the most disturbing symptom and chief complaint in IDD cases. Lack of early signs impairs the ability of early identification of IDD. Thus the disease is commonly irreversible at a later stage. These remind us that more in-detail studies into the common pattern of human IDD development are needed. Encouragingly, some recent studies combined new technology, including single-cell RNA-sequencing, with human specimens to discover the disease's very nature. Recent studies by Gan et al. (135), Gao et al. (136), Han et al. (137) and Zhang et al. (138) made delightful exploration into the possible reason for IDD initiation. Subsequent studies are needed to determine the similarities and differences between patients with different natural disease histories.

In developing appropriate animal models, some important considerations need extensive attention. Firstly, the upright position determined the unique mechanical property of the human spine and intervertebral disc. Secondly, the notochordal cells undergo apoptosis and differentiation after birth and are absent in the adult human spine. But notochordal cells may remain in the intervertebral discs in certain specimens, which may promote the regeneration ability of damaged discs. Thirdly, the duration between modeling and detectable degeneration should be taken into consideration. If the degeneration occurs too soon, it is unlikely to replicate the actual circumstances in IDD. Severe structural destruction will conceal the effectiveness of certain therapy. Lastly, the ethical and cost issue should also be taken into consideration.

In this review, we elaborated on the commonly used method to construct IDD models, which mainly fall into three categories: damage-induced, mechanical, and spontaneous. Damage-induced models make punctures or incisions into the intervertebral discs and impair the integrity of the disc structure, while mechanical models exert external force into the disc and accelerate the degeneration process. The spontaneous models focus on common IDD causes, such as aging and collagen loss, which spontaneously lead to IDD development. Each category replicates a certain stage of IDD to some extent. Therefore, in searching for possible treatments for IDD, we should emphasize the importance of selecting correct animal models. For example, SPARC-null mice develop significant chronic back pain, making it suitable for researching IDD-related pain. The discectomy model mimiked the clinical situation of disc resection and seemed ideal for developing disc regeneration therapy. Integrating more than one IDD animal model into one study is becoming more common (139). Combining these models is a helpful approach to gaining solid evidence for the efficacy of specific interventions.

## 4. Conclusions

In conclusion, animal models are indispensable for understanding, characterizing, and treating disc degeneration. However, despite the methods listed in this review, there is still no consensus on which model best mimics IDD. More importantly, there is still some gap between model-induced IDD and actual clinical features. Further studies are needed to determine the fidelity of these models and eventually contribute to developing new IDD therapeutic strategies.
